# The oxytocinergic system mediates synchronized interpersonal movement during dance

**DOI:** 10.1038/s41598-018-37141-1

**Published:** 2019-02-13

**Authors:** Liad Josef, Pavel Goldstein, Naama Mayseless, Liat Ayalon, Simone G. Shamay-Tsoory

**Affiliations:** 1 The Graduate School of Creative Arts Therapies, University of Haifa, 199 Aba Khoushy Ave. Mount Carmel, Haifa, 3498838 Israel; 2 Department of Psychology and Neuroscience, University of Colorado, Muenzinger D244, 345 UCB Boulder, Boulder, CO 80309-0345 USA; 3 Department of Psychology, University of Haifa, 199 Aba Khoushy Ave. Mount Carmel, Haifa, 3498838 Israel; 4 Department of Psychiatry and Behavioral Sciences, Stanford University, Stanford, USA

## Abstract

Because the oxytocinergic (OT) system has previously been linked to regulation of complex social cognition and behavior, we examined whether intranasal administration of OT would modulate synchronization during a real-life dance paradigm. The current study examined pairs of friends while dancing after intranasal administration of OT or placebo. Motion tracking software and a computational model were utilized to measure synchrony between the partners as manifested in the velocity of their movements. In line with our predictions, OT increased synchrony between partners. This effect was stronger for individuals with higher trait empathy scores. We concluded that the OT system plays an important role in promoting interpersonal synchrony during dance, suggesting that OT underlies the kinesthetic dimension of empathy. Although the biological mechanisms underlying empathy have been studied extensively, scientifically validated knowledge about the kinesthetic dimension of empathy is still lacking. The current study supports the hypothesis that interpersonal synchronization in body movement could be a marker of kinesthetic empathy.

## Introduction

The human ability to synchronize with other individuals has important evolutionary roots^1^. Examples include synchronized periodic movements to generate acoustic signals^2–4^, synchronous flashing among fireflies^5^, synchronized collective movements among predators while hunting^6^ and synchronized reactions to stressful and dangerous situations^6,7^. Humans also show a tendency to imitate the postures or actions of others^1,8,9^. This capacity develops early in life^10,11^ and plays a key role in the development of infant-mother bonding and in social communication in general^12,13^. For example, synchronized behaviors have been reflected in speech understanding^14^ and in psychotherapy^15^, indicating that motor synchrony plays a major role in affiliative behaviors and in the development of social behavior.

Nonverbal synchrony can serve as an indicator of different aspects of social interaction^15–17^. Bernieri and Rosenthal (1991) suggested that the degree of rapport between people is reflected by the behavioral synchrony between them^1^. In other words, individuals tend to synchronize their behaviors to a greater degree when interacting with people they like.

Dance is thought to have evolved in humans in order to increase interpersonal cooperation, feelings of togetherness and group cohesion. Indeed, most forms of joint dance involve synchrony^18,19^. Due to the general importance of dance in human life, and specifically to its ability to serve as a healing and therapeutic approach^20^, in the current study we chose to examine dance as an example of the synchrony that occurs naturally under ecological conditions of nonverbal movement interactions. Given the role of the neurohormone oxytocin (OT) in regulating social behavior, we sought to examine whether OT modulates interpersonal synchrony during dance. OT, a neuropeptide produced in the hypothalamus, has become the target of a large number of studies that have demonstrated its influence in various aspects of social behavior (for review, see^21^). Initial research on non-humans has demonstrated the role of OT in maternal behavior^22^, mating^23,24^ and social recognition^25^.

Some researchers have suggested that OT is related to the regulation of complex social cognitions and behaviors in humans^26^. Others have proposed that OT plays a key role in reducing anxiety and in relaxation, growth and restoration^27^, while still others have suggested that OT induces a general sense of well-being and improves social interactions^28^. Relevant to our current research, OT was found to play an important role in synchronized behaviors and interactive reciprocity in mother-infant relationships^11^, as well as in synchronized pair states in romantic relationships^29^, social coordination^30^, joint action^31,32^ and empathic abilities^20^.

**Figure 1:**
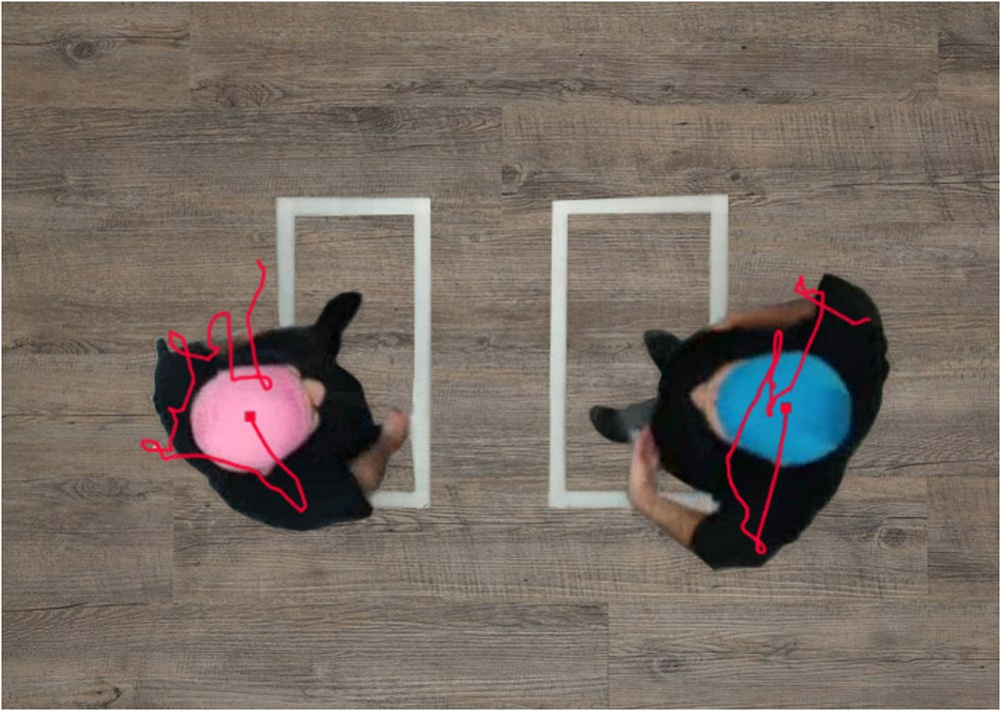
Dancing pairs. The rectangles on the floor mark the movement borders.

Accordingly, we designed an ecological experiment in which pairs of friends were asked to move freely around a room either together or separately, and we explored their tendency to synchronize their movements with one another following intranasal intake of either OT or placebo (Fig. 1). Moreover, since a previous study showed that OT promotes the choice of closer interpersonal distances among highly empathic individuals^33^, we hypothesized that OT administration (vs. placebo) would have a larger effect in dyads that exhibit increased trait empathy. Empathy has been theorized and studied mainly in terms of emotional and cognitive understanding of another person’s emotional states^34,35^, while only a few studies have explored bodily kinesthetic empathy. Kinesthetic empathy is an important aspect of empathy in that it refers to an individual’s own corporal feelings and awareness of the sensation of movement in response to someone else’s body movements or postures^36^. Recent research demonstrated that OT and empathic ability have a genetic connection^37^ and that empathic difficulties may underlie impairments in social and interpersonal skills, resulting in lower motor synchrony^38^. Thus, we suggest that highly empathic individuals should reflect higher motor synchrony after OT administration.

## Results

The OT and placebo groups did not differ in age [t(59) = −0.54, p = 0.59], marital status [t(59) = −0.51, p = 0.60] or relationship status (in a romantic relationship or not) [t(59) = −0.55, p = 0.59]. The sample consisted of 83.6% single male participants and 16.4% married male participants. Of the single participants, 37.7% were not currently in a romantic relationship, while 62.3% were. Both partners in 48% of the dyads reported that their friend (partner in the study) was among their five best friends. This information, however, was not related to the level of movement synchrony (r(30) = 0.03, p = 0.88). There was no difference in intimacy level between the OT and Pl groups (t(30) = 0.44, p = 0.66), though movement synchrony showed a significant correlation with intimacy level (r = 0.40, p = 0.024).

To ensure that our synchrony measurement was not a result of artifacts, we first compared the two dance conditions based on a within-subject design: individual vs. paired conditions. For this analysis individual data were paired to the same dyads as in the paired condition and the velocity synchrony was then calculated as described in the methods section. A paired-sample t-test revealed significant differences between velocity synchrony in the individual and the paired conditions [t(30) = 12.99, p < 0.0001, Cohen’s D = 4.74], with higher synchrony in the paired condition (M = 0.62, SD = 0.18) than in the individual condition (M = 0. 27, SD = 0.11).

**Figure 2:**
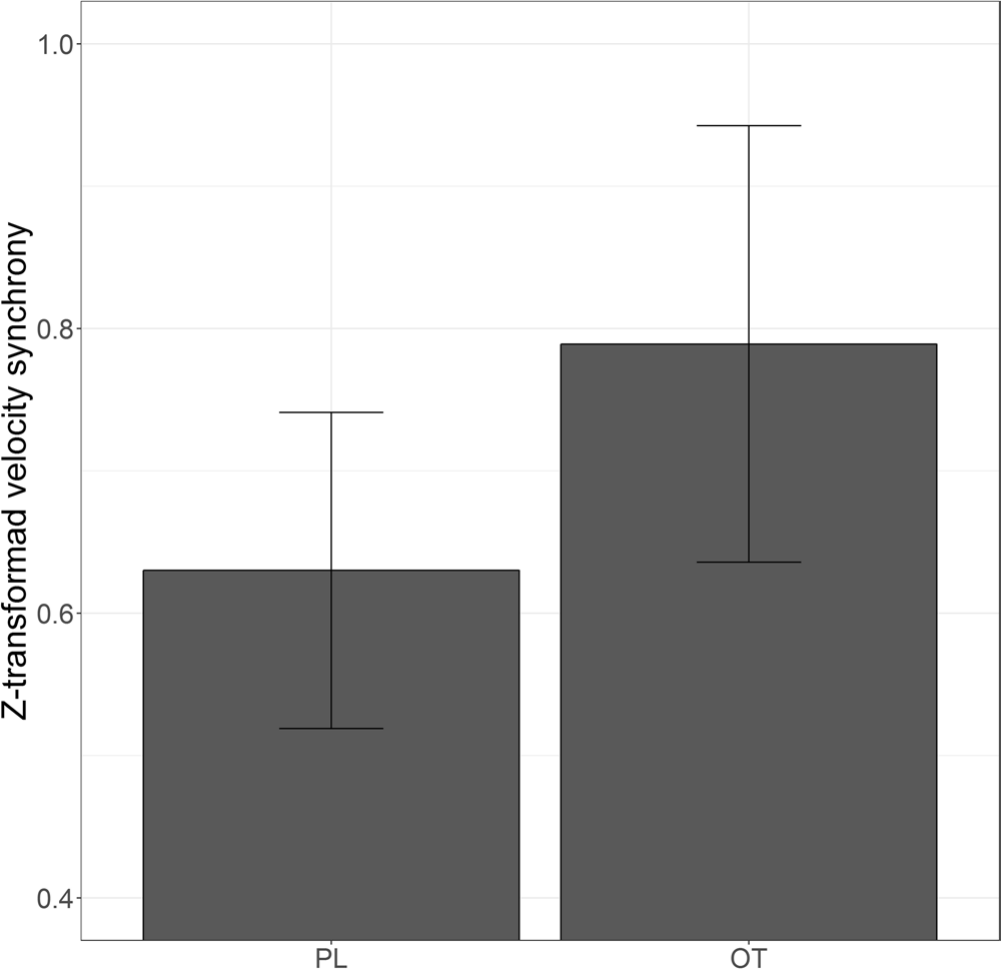
Head movement velocity synchrony in OT vs. placebo with CI 95%.

An independent-sample permutation t-test was conducted to test the main hypothesis regarding differences between OT and placebo intake under maximum synchronization. A significant difference was found between OT and placebo [t(29) = 2.31, p = 0.017, Cohen’s D = 0.90], showing higher synchrony for OT (M = 0.79, SD = 0.20) than for placebo (M = 0.63, SD = 0.14) (see Fig. 2). As an additional cross-validation of the results, OT and placebo head velocity synchrony were compared in the individual condition using permutation testing. The results revealed no significant findings [t(29) = 0.62, p = 0.54], validating that the synchrony measurement was not a result of random sources of synchrony.

**Figure 3:**
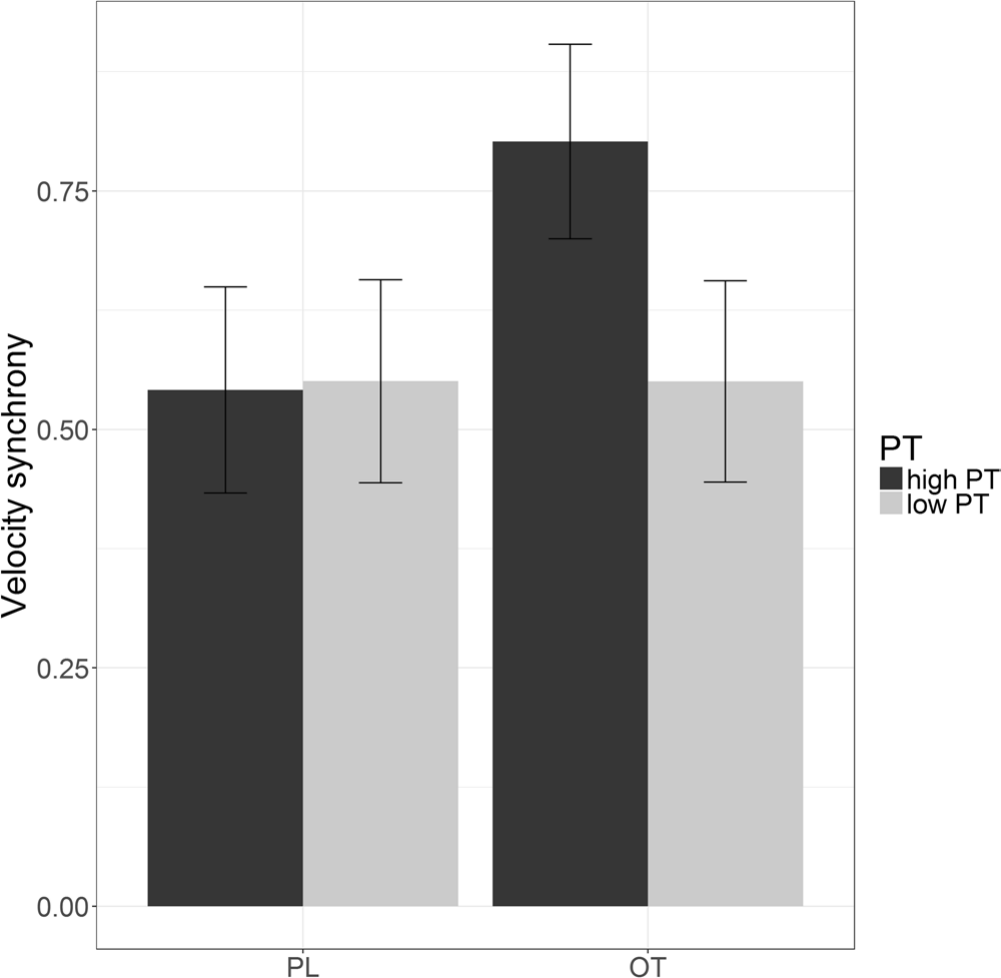
Perspective-taking moderates the effect of oxytocin on velocity synchrony. OT = oxytocin, PL = placebo, PT = Perspective Taking. Confidence intervals 95% is included.

A linear regression was used to test the secondary hypothesis that the effect of OT in increasing synchrony would be greater for those who rank high on empathy trait and state. The results indicated that OT manipulation significantly moderated the relationship between the pairs’ average score on the Perspective Taking (PT) subscale of the Interpersonal Reactive Index (IRI) and their velocity synchronization [F_(1,27)_ = 5.83, p = 0.023, ΔR^2^ = 0.13] (Fig. 3). The dyads with low PT exhibited no effect of OT on head movement velocity synchrony (B = 0.0003, t_(27)_ = 0.005, p = 0.99), while the dyads with high PT exhibited an elevated effect of OT on head movement velocity synchrony (B = 0.26, t_(27)_ = 3.43, p = 0.002, Cohen’s D = 0.93).

The same pattern of moderation also emerged for the two state empathy self-report markers: [F_(1,27)_ = 13.94, p < 0.001, ΔR^2^ = 0.28] for self-perspective and [F_(1,27)_ = 5.26, p = 0.029, ΔR^2^ = 0.14] for other-perspective, respectively. For the dyads with low-state empathy, OT exhibited no effect on head movement velocity synchrony (self: B = −0.07, t_(27)_ = 0.88, p = 0.35; other: B = −0.03, t_(27)_ = 0.33, p = 0.79), while for the dyads with high state empathy OT exhibited an increased effect on head movement velocity synchrony (self: B = 0.36, t_(27)_ = 4.52, p < 0.001, Cohen’s D = 0.93; other: B = 0.36, t_(27)_ = 3.27, p < 0.001, Cohen’s D = 0.93). The interactions with the other IRI subscales were not significant.

## Discussion

The current study sought to examine the role of the OT system in modulating synchrony during dance. We measured the synchrony that naturally occurs between pairs of friends under the influence of intranasal OT vs. placebo. As hypothesized, administration of OT, but not placebo, increased movement synchrony in dancing pairs.

These results are consistent with previous studies reporting the role of OT in synchronized behavior and interactive reciprocity^11,29^ as well as in the regulation of complex social cognition and behaviors^26^. The results are also in line with studies suggesting that intranasal administration of OT may enhance social synchrony and coordinated behaviors^30–32^, as well as improve different aspects of social interaction (see^21,28^ for reviews). Many researchers have suggested that nonverbal coordination and body movement synchrony may serve as a measurable index for aspects of interpersonal interaction^1,15–17^. Therefore, in the current study we postulated that synchronized movement may be a central aspect of kinesthetic empathy.

OT has been shown to alter the perceptual salience of social cues^39,40^. The current study adds to this finding by suggesting that OT promotes *kinesthetic cues* that foster interpersonal synchrony. The secondary hypothesis of our study was that OT would increase synchrony in dance to a greater degree for people with higher empathic abilities. This hypothesis was confirmed for the PT IRI subscale of empathy, which assesses the tendency to spontaneously adopt the psychological point of view of others. This tendency is considered to be a cognitive component of empathy, akin to “theory of mind”^35^. In the current study, a greater degree of synchrony was observed among individuals who were better able to adopt the psychological point of view of others. This is consistent with the notion that empathic abilities may shape the way individuals process social stimuli^33^ and with findings that OT increases sensitivity to social cues depending on inter-individual factors^39^. It is also consistent with research showing that a cognitive-motor process is involved in rhythmic interpersonal coordination, enabling individuals to represent joint action goals and to anticipate, attend to and adapt to the actions of others in real time^41^. This cognitive-motor system is further known to be influenced by social psychological factors such as empathy^41^. Hence, it is possible that OT enhances the expression of empathic abilities, thus translating trait empathy into state empathy. In line with this, research has reported that OT increases emotional empathy^30,42^. In the current study we examined a possible connection between situational (state) empathy—that is, empathic reaction in a specific situation—and dispositional (trait) empathy, where empathy is understood as a person’s stable character trait. Since the mechanisms of trait empathy are reflected in behavior (state empathy) it is possible that OT increases state empathy in participants with high trait empathy. This can be explained through an analogy to gene expression: trait empathy is like a DNA sequence and state empathy is like a protein decoded from the corresponding DNA. OT here acts as catalyst for enhancing creation of the protein.

It is important to mention that while traditional paradigms examining empathy usually involve computerized paradigms, here we explored a type of empathic behavior that occurs naturally under ecological conditions of nonverbal interaction in movement and dance. The use of an automated and objective measure of synchrony in this study makes an important contribution in view of the increasing need for computational approaches for annotating, evaluating and modeling interactional synchrony.

Moreover, the current study sheds light on the importance of nonverbal kinesthetic cues related to empathy and attunement and has important implications for any human interaction. A better understanding of the neurobiology of human social interactions can make a significant contribution to the development of novel clinical approaches to mental disorders associated with social deficits. It is important to note that while we addressed kinesthetic empathy as a theoretical framework for synchrony, scientifically validated knowledge on the embodied and kinesthetic dimensions of empathy is still lacking^36^. Recent research shows that kinesthetic empathy is critical for human well-being^19,43,44^. Nevertheless, future studies are warranted to examine this topic further as well as to develop validated measurements of kinesthetic empathy.

Although the current study used a controlled design, it has several limitations that need to be acknowledged. First, our sample contained only male participants due to potential artifacts of OT manipulation in female participants. Therefore, future studies are needed to try to replicate our findings for women. Second, our sample size is not very large, even though our post-hoc power analysis validates our findings (for both hypotheses power >0.80).

Because empathy is strongly correlated with positive outcomes in psychotherapy^45–47^ and because the potential of intranasal OT as a therapeutic agent in psychiatry is growing^48^, our findings may aid in devising therapeutic interventions for enhancing empathy. Moreover, the objective tool utilized in our study for measuring interpersonal synchrony can be implemented as a method for monitoring the empathic aspects of therapist-client relationships^49^. As established in a review evaluating interpersonal synchrony methods across disciplines^50^, the lack of automated tools for studying synchrony has limited the exploration of psychiatric conditions that affect social abilities, whether permanent (e.g., autism) or temporary (e.g., major depression). Due to the growing potential of intranasal OT as a therapeutic agent in psychiatry, it is important to understand the distinctive effect of this substance in relation to personal schemas such as empathic abilities.

## Materials and Methods

### Participants

Sixty-two healthy male participants (mean age = 26.29, SD = 3.4) comprising 31 pairs of friends were recruited through advertisements posted throughout the University of Haifa and on relevant websites. Female participants were excluded from the current study to prevent hormonal interactions between OT and estrogen^43^. The pairs were randomly assigned to receive either OT or placebo. In exchange for their participation, participants received monetary compensation of 100 ILS. The study was approved by the Faculty of Social Sciences Ethics Committee, University of Haifa. All participants gave their written informed consent for participation and all methods were carried out in accordance with relevant guidelines and regulations.

### OT/placebo administration and procedures

The experiment was based on a double-blind design. Participants in each dyad randomly received either 24 international units of intranasal OT (Syntocinon spray, Defiante, Sigma) or 24 IU of sterile saline as placebo treatment (PL). The placebo consisted of the same saline solution in which the hormone was dissolved but without the hormone itself. Both treatments were self-administered using a nasal spray, three puffs per nostril, with each puff containing 4 IU. Neither the experimenter nor the participant knew whether the participant was receiving OT or the placebo, but session order was randomized such that half the participants received OT in the first session and the other half received OT in the second session. Following treatment, participants were asked to wait 45 minutes from the time of administration to ensure that the OT levels in the central nervous system reached a plateau^51^. During this time, participants were given a nature journal to read. At the end of these 45 minutes, the participants were debriefed again, and the experiment began.

### Experimental task

The task included two conditions presented in counterbalanced order: a *pair condition* and an *individual condition*. In both conditions participants were instructed to move freely and dance using any style, rhythm and movements they pleased while remaining inside a designated zone in the room. In the *pair condition*, participants were placed facing each other and were instructed not to talk to or touch one another. In the *individual condition*, participants were instructed to move freely around the room by themselves. The *individual condition* served as a baseline measure of basic movement, allowing comparison between random-source synchrony (data derived from a timed series of each participant dancing separately) and real pair synchrony (data derived from a timed series of the *pair condition*, i.e., dancing together).

### Motion Tracking

Participants were placed in a designated zone in the room where their movements could be recorded by a camera. In the *pair condition*, participants were placed facing each other, separated by a distance of 60 cm (Fig. 1).

A ceiling-mounted camera recorded participants’ head movements, providing 30 frames per second of data. Ethovision-XT 9Motion Tracking Analyze software was used to measure head movement velocities. Offline, the Ethovision software defined the locations of the participants by color-tracking caps of different colors placed on the participants’ heads. Blue and pink caps were chosen as they provided the best contrast against the setting background. Automatic tracking was followed by manual examination of outliers, specified as motions detected outside the cap parameters. These were defined as errors and were deleted from further analysis. Linear interpolation was used to infer continuous motion. The velocity of each partner was defined as the distance moved by the participant’s center point per time unit. Since the camera was placed above the participants, the most of variability was represented by two space dimensions parallel to the floor.

A recent study showed that therapeutic success is associated with head synchrony between a psychotherapist and a patient^52^, thus demonstrating the potential of head synchrony to serve as a marker of interpersonal communication quality.

### Synchrony measurements

Synchrony of the head motions of dyads was used as a marker of interpersonal synchrony, based on previous studies using automatic techniques for measuring synchrony of velocity. Those studies chose to focus on head motions as the head plays an active role in interpersonal interaction and can express emotion and acknowledgement (for review, see^1^). Head motion has also been used to analyze nonverbal pair interactions in conversations (for review, see^50^) and in pair dancing^53^. In the current study, the participants’ head movements were tracked via Ethovision video tracking software, providing real-time velocity output for each head. We analyzed the inter-partner relationship in the velocity data based on time-lagged cross-correlations. Since the dynamics of the interactions between the dyads may differ, for each dyad we used 8-second windows with running steps of 1 second to identify the lag closest to zero that shows maximum correlation. Application of windows of 2, 6, 8, and 10 seconds yielded similar results. After this lag was identified, the Fisher’s Z transformed values of the cross-correlations were averaged.

### Validation of the synchrony measurement

**Figure 4:**
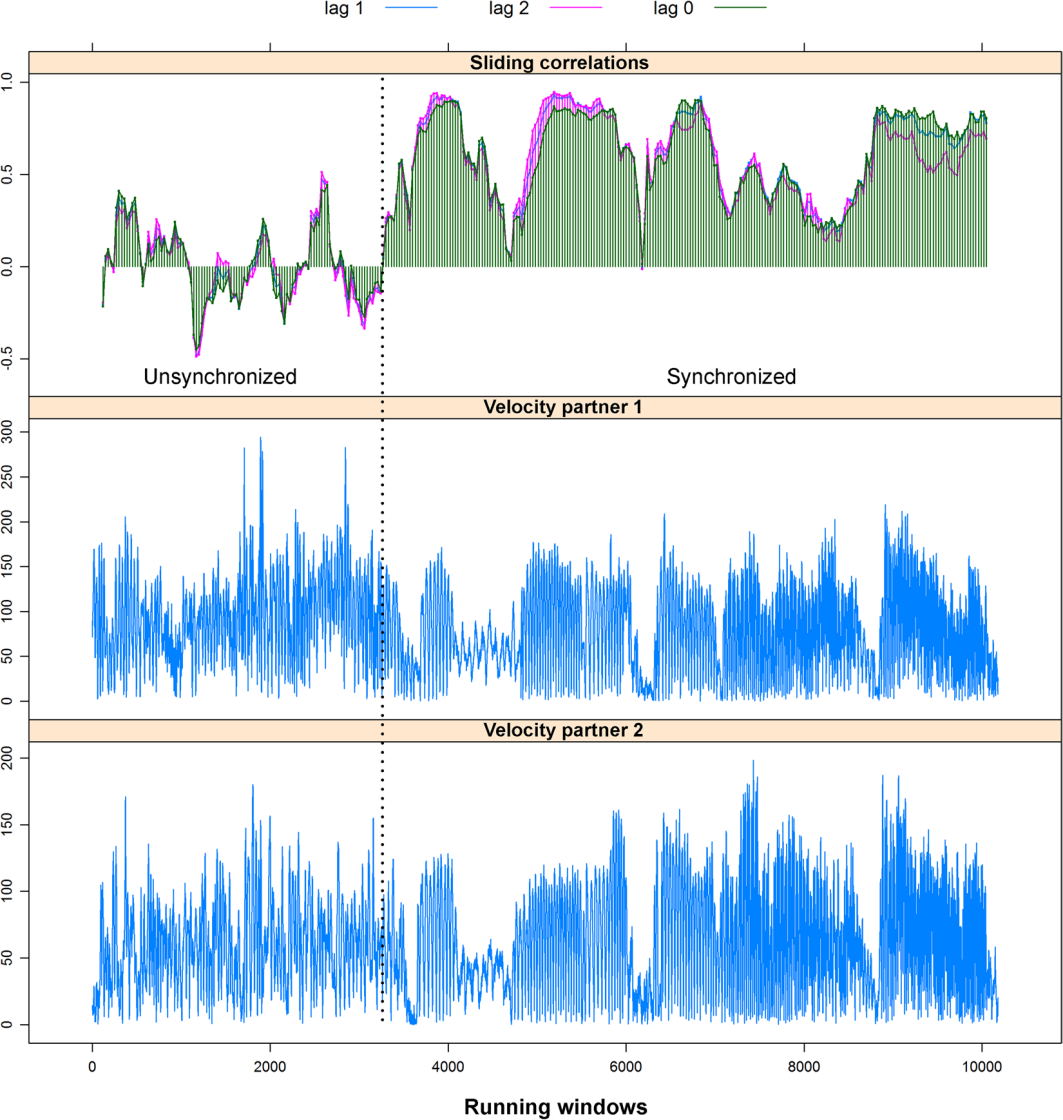
Simulation trial of head motion synchrony. The two bottom plots show the changes in velocity over time. The upper plot shows the sliding window correlation for a window width of 8 sec. and step of 1 sec. The dotted line shows the participants who were asked to synchronize their movements. As expected, the unsynchronized movements show low correlations, while the synchronized movements demonstrate high and consistent correlations.

Prior to the current study, we validated our synchrony measures though a simulation in which one pair was first asked to move asynchronously and afterwards synchronously. The proposed synchrony analysis was applied to the data output from the Ethovision tracking software. Figure 4 shows the running window cross-correlations for the best three lags, confirming that low correlations were evident in the non-synchronized dance, while high correlations appeared in the synchronized dance.

### Empathy and intimacy measurements

The Interpersonal Reactive Index (IRI)^54^ was employed to measure empathic abilities. The IRI consists of four seven-item subscales: Perspective Taking, Fantasy, Empathic Concern and Personal Distress. The internal reliabilities of all four subscales were substantial (coefficient α ranging from 0.71 to 0.77), as was the test-retest reliability (r’s ranging from 0.62 to 0.80 over a 10-week period). The scales were validated with other measures of empathy, sensitivity to others and intellectual abilities, as well as with interpersonal functioning measures assessing a broad range of social behaviors^54^.

At the end of the session each participant was asked to rate the following statements reflecting state empathy on a scale ranging from 1 to 7: “It’s easy for me to see things from his point of view right now” (self-perspective); “It’s easy for him to see things from my point of view right now” (other-perspective). The partners’ intimacy level was measured by the Intimate Friendship Scale (32 items) developed by Sharabani^55^.

### General procedure

After the participants arrived and signed informed consent forms, they were given an intranasal dose of either OT or placebo. Each member of the pair was then taken to a separate waiting room, where they were kept apart for 45 minutes—the time needed for the hormone to reach plateau levels in the brain. After that, the members of the pair were both escorted into a private room where they participated in the two 5-minute freestyle dancing conditions—the *individual condition* and the *paired condition*—using across-subject counterbalancing. Both conditions took place without music, and participants were asked to wear colored head caps used to track their head positions offline (Ethovision-XT). A camera located in the ceiling above the participants recorded their movements. After the participants confirmed that they understood the task, the experimenter left the room and the recording began.

### Data Analysis

All statistical analyses were conducted using R 3.3.3 software^56^. The level of significance was α = 0.05 (two-tailed). An independent-sample permutation test (1000 permutations) was used to test the primary hypothesis regarding the effect of OT on velocity synchrony^57^. Linear regression was used to test the secondary hypothesis that the effect of OT in increasing synchrony would be greater for those who rank high on empathy traits and lower for those exhibiting higher anxiety. To interpret the interactions, we used a model-based contrast analysis to compare OT/PL group conditioning on the level of the moderators (low level = 1, SD below the mean; high level = 1 SD above the mean). For both hypotheses, simulation study was used to calculate the power of the significant findings. After synthetic data were generated 1000 times using the obtained parameter estimates, the initial analysis was conducted. The power was computed by comparing p-values from the simulated data to the defined type-one error (0.05).
